# Microwave-Assisted Base-Free Oxidation of Glucose with H_2_O_2_ on Gold- and Manganese-Containing SBA-15—Insight into Factors Affecting the Reaction Pathway

**DOI:** 10.3390/ijms23094639

**Published:** 2022-04-22

**Authors:** Izabela Sobczak, Tsering Chödon Kowalska, Magdalena Nowicka, Maria Ziolek

**Affiliations:** Adam Mickiewicz University, Poznan, Faculty of Chemistry, Uniwersytetu Poznanskiego 8, 61-614 Poznan, Poland; tsewas@st.amu.edu.pl (T.C.K.); magdalena.nowicka682@gmail.com (M.N.); ziolek@amu.edu.pl (M.Z.)

**Keywords:** gold-manganese SBA-15, Au dispersion, microwave-assisted glucose oxidation with H_2_O_2_, base-free oxidation, reaction pathway, effect of Lewis acid sites and pore size, activation energy

## Abstract

The aim of this work was to gain insights into the role of manganese in MnSBA-15 support for gold in the base-free glucose oxidation with H_2_O_2_ using a microwave reactor. MnSBA-15 (manganese—acidity source) and SBA-15 (for comparison) were modified with Au (2.2 wt. %) and Cu (for comparison). The physicochemical properties of the catalysts were investigated by XRD, N_2_ ads/des, TEM, UV-vis, XPS, pyridine adsorption combined with FTIR, ATR-FTIR, and 2-propanol decomposition. The effects of the Mn presence in the support, Au NPs size that determines the number of active Au centers, and the Fermi energy (E_F_), together with the effects of the pore size, reaction temperature, and time on the activity and selectivity of the applied catalysts were assessed and discussed. It has been demonstrated that the presence of Mn generated Lewis acid centers which did not participate in glucose and H_2_O_2_ adsorption, and thus, were not directly involved in the reaction pathway. Both reagents were adsorbed on gold nanoparticles. H_2_O_2_ was decomposed to molecular oxygen which oxidized glucose to gluconic acid (50–90% of glucose conversion depending on the reaction time and ~100% selectivity). The presence of manganese in MnSBA-15 was responsible for increased Au NPs size and only slightly influenced the negative charge on gold particles. To achieve effective activity a compromise between the number of active gold species and the level of E_F_ has to be reached (for 5.7 nm Au NPs).

## 1. Introduction

The oxidation of sugars, which can be easily obtained from the transformation of biomass, has recently attracted considerable attention [1,2]. The main focus in this area is on glucose oxidation to gluconic acid, obtained by the oxidation of the aldehyde group to the carboxyl group at carbon-1 (C-1) of glucose [3]. This target product and its derivatives are generally used in the food industry as acidity regulators, and as additives in the pharmaceutical, building, and textile industries [4]. Au-based catalysts (e.g., Au/C, Au/Al_2_O_3_, Au/TiO_2_) [4,5,6,7,8,9,10], Au-zeolites, and Au-mesoporous silicas [4,11,12,13]) were found to be active and selective for the oxidation of glucose to gluconic acid under basic and base-free conditions. Molecular oxygen has been most often used as an oxidant in this reaction. However, hydrogen peroxide (also a green oxidant) is a valid alternative to oxygen. The advantage it offers is its complete solubility in aqueous media even at high concentrations, which excludes any possible mass transfer limitation [2].

Microwave-assisted catalytic reactions have gained much attention in recent years as a powerful tool for rapid and efficient synthesis of a variety of compounds [14]. Microwave irradiation-assisted chemical transformations are pollution free, eco-friendly, and occur with high yields and offer simplicity in processing and handling. The application of microwave dielectric heating allows the temperature increase to be uniform throughout the sample (catalyst, solvent, or reagent), leading to fewer by-products and/or product decomposition. The use of microwave energy often results in good yields in a short time as compared with those of the reactions by classical synthetic methods. Due to the short reaction time and expanded reaction range, microwave synthesis meets the high demands imposed on the chemical industry and permits obtaining required reactivity and selectivity of reactions. This method is a practical alternative to conventional heating [14,15]. Moreover, the combined use of microwave irradiation and heterogeneous catalysts gives an additional benefit of easy separation of the catalyst and products. It is important to note, that to the best of our knowledge, there are only a few studies dedicated to microwave-assisted glucose oxidation [9,10,13].

The mechanism of microwave-assisted glucose oxidation on gold-containing catalysts in the presence of hydrogen peroxide has not yet been clearly elucidated. The current literature does not provide exhaustive answers to the questions about possible reaction pathways in the glucose oxidation reaction. One of the fundamental questions is the reaction pathway and the kind of active centers involved in chemisorption/transformation of hydrogen peroxide and glucose, as well as the further interaction between the chemisorbed species. According to Kiyonaga et al. [16], hydrogen peroxide is chemisorbed on Au surface active species and such chemisorption is accompanied by decomposition of H_2_O_2_ to OH^−^ ions and HO^•^ radicals. Hydroxyl ions react with free H_2_O_2_ forming HO_2_^−^ and H_2_O. Finally, HO_2_^−^ species react with HO^•^ and O_2_ and H_2_O is formed. The higher the electron density on the surface of Au NPs, the faster the electron transfer in the first step of this redox pathway. For base-free microwave-assisted glucose oxidation with hydrogen peroxide on Au/Al_2_O_3_, Rautiainen et al. [9] have shown a correlation between the rate of oxygen formation and the activity of gold catalysts. Walkowiak et al. [13] have suggested that the rate limiting step in microwave-assisted glucose oxidation with H_2_O_2_ performed on gold-modified zeolites is chemisorption and decomposition of the oxidant. Moreover, the authors concluded that there is no correlation between BrØnsted acid sites (BAS—strength and/or number) of the zeolite support on which glucose can be chemisorbed and the activity in glucose oxidation with H_2_O_2_, in contrast to the oxidation of glucose with molecular oxygen in which the enhancement in glucose conversion was related to some extent to the increase in glucose chemisorption on BrØnsted acid sites (BAS). However, Lewis acid sites (LAS) in zeolites have been also identified as capable of selective adsorption of sugars from aqueous solutions [17]. Thus, glucose can be chemisorbed on LAS with the participation of water and the formation of water-glucose-LAS complex, as proposed in [12]. It has been suggested that the contribution of LAS in the reaction pathway is associated with the adsorption of glucose molecule via formation of coordinative bond between LAS and oxygen from hydroxyl group at C6 or carbonyl oxygen (at carbon atom C1). In the next step, active oxygen species adsorbed on Au NPs oxidize the adsorbed glucose molecules. Nevertheless, such a possibility has not been considered in the literature in the context of microwave-assisted glucose oxidation with H_2_O_2_. Therefore, to answer the question about the possible role of LAS in this reaction, the gold support containing exclusively Lewis acid centers (without BAS) has been applied in this work. For preparation of the support containing Lewis acid sites, we were looking for a transition metal that would have a relatively low acidity strength so as not to chemisorb glucose and/or the intermediate too strongly, as was the case, for example, with the niobium modifier [11]. Based on literature data [18] showing that Lewis acidity was lower for MnSBA-15 than, e.g., for ZrSBA-15, in this work Mn was chosen as a modifier of SBA-15 support.

The idea of this study was not only to use the gold support containing LAS without BAS, but also to construct catalysts with different textural parameters (especially different pore sizes) but with the same elemental composition and structure. Recently [13] it has been established that for the same gold particle size the activity of Au/MCM-36 in base-free and microwave-assisted glucose oxidation with hydrogen peroxide was higher than that of Au/HBeta zeolites. This was attributed to the larger pores in the first zeolite. However, the structure of both compared catalysts was different, and therefore, not only the pore size but also the structure could have an impact on the activity. Therefore, in this work the same SBA-15 structure with different pore sizes was applied. To get different pore sizes in hexagonally ordered mesoporous silica, SBA-15, two various manganese sources (Mn(II) nitrate and acetate) and slightly different preparation procedures were used in the synthesis of MnSBA-15. The use of MnSBA-15 molecular sieves as supports for gold assumed the possible interaction of gold and manganese species and in this way modification of gold particle size and electronic properties. Therefore, the results obtained for this system are expected to answer the question about the role of accumulation of negative charge on gold nanoparticles (AuNPs), postulated as one of the important features in chemisorption and transformation of hydrogen peroxide [13] and the catalyst activity. The impact of manganese dopant on the size of Au NPs and the catalyst activity would also be considered.

## 2. Results and Discussion

### 2.1. Characterization of Catalysts

#### 2.1.1. Composition and Structural/Textural Properties

Table 1 shows the content of metals (Mn, Au) in the catalysts prepared obtained from ICP-OES measurements. The results indicate that the actual content of manganese in the samples, expressed as wt. %, was much lower than assumed (Section 3.2). The loading of Mn was ≤0.5 wt. %. This indicates the difficulty of Mn introduction in higher amounts into SBA-15 structure during hydrothermal synthesis (despite lowering the acidity (pH = 3) of the synthesis mixture). Interestingly, the loading of Mn was 2.5 times higher for Mn(N)SBA-15 (N denotes manganese(II) nitrate used as metal precursor) than Mn(A)SBA-15 (A denotes manganese(II) acetate used as metal precursor), indicating the effect of metal precursor and synthesis conditions.

Gold loading in mesoporous silicas modified by grafting with organosilane was as expected (2.1–2.2 wt. %). It confirms the earlier results [11,19,20] showing that the interaction of gold (in the form of AuCl_4_^−^ ions [21]) with NH_2_ groups (in the form of NH_3_^+^ ions [21]) is effective for gold incorporation on the silica surface. However, this modification method influences the manganese content in the samples. The loading of Mn in both materials modified with gold was lower than before gold introduction (Table 1). The high decrease in Mn content as a result of gold modification was observed for Mn(A)SBA-15 (a decrease by 35%). This proves the partial removal of manganese species present in Mn(A)SBA-15 during the two-step modification (grafting with (3 aminopropyl)trimethoxysilane (APTMS) in toluene and gold anchoring). For Au-Mn(N)SBA-15 the decrease in manganese content was much lower (only 8%). This indicates the presence of more stable manganese species in this sample than in Mn(A)SBA-15.

**Figure 1:**
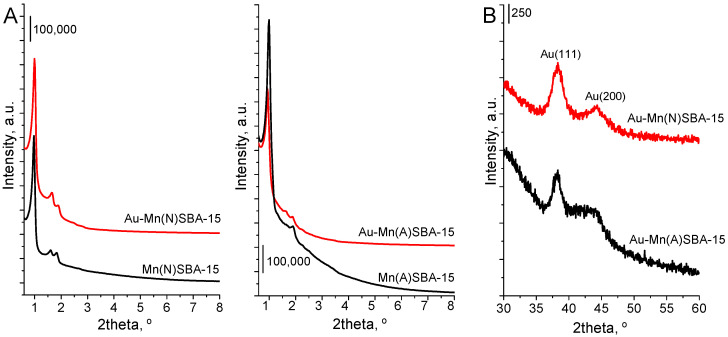
XRD patterns of MnSBA-15 and Au-MnSBA-15 materials. (**A**) The range of 0.6°–8° 2θ; (**B**) the range of 30°–60° 2θ; typical of X-ray diffraction peaks of Au(111) and Au(200) planes.

XRD and N_2_ ads/des studies were performed in order to investigate the structural/textural properties of SBA-15 catalysts. The small angle XRD measurements were performed in order to confirm SBA-15 structure of mesoporous silica after introduction of manganese during the synthesis and after post-synthesis modification with gold. The XRD pattern of Mn(N)SBA-15 support (Figure 1A) exhibited three typical and well resolved diffraction peaks (2 theta at ca. 1°, 1.6° and 1.8°) assigned to the faces (1 0 0), (1 1 0), and (2 0 0), which clearly evidenced the hexagonal ordered mesoporous structure with space group of P6mm [22]. For Mn(A)SBA-15, only the reflections at 2theta = 1° and 1.8° appeared in the XRD pattern (without that at 1.6°) (Figure 1A). This is probably due to the presence of manganese species (bulk oxides) in pores of mesoporous silica which disturb the ordering of pores. Both the reflections (at 2theta = 1.6° and 1.8°) are present in the diffractogram of Au-Mn(A)SBA-15 as a result of the partial removal of manganese species during the procedure of modification with gold.

**Figure 2:**
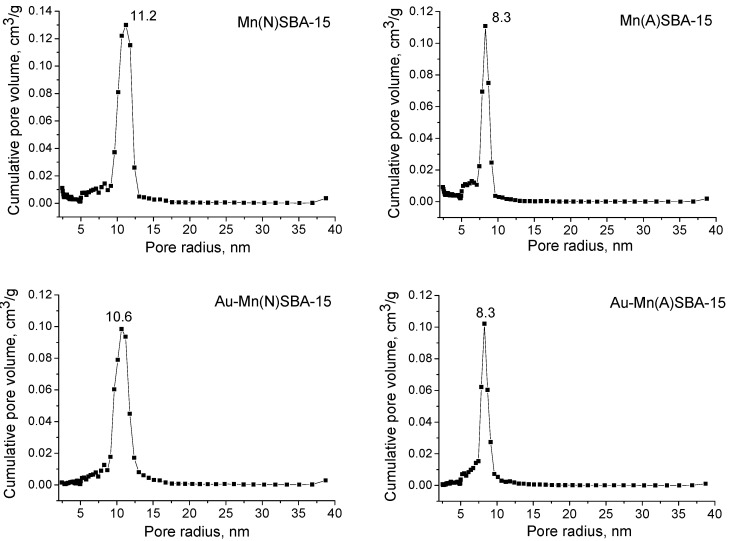
Pore size distributions for MnSBA-15 and Au-MnSBA-15 materials.

The mesoporous character of the samples prepared was confirmed by the N_2_ adsorption/desorption isotherms (Figure S1). The isotherms of Mn(N)SBA-15 and Mn(A)SBA-15, before and after modification with gold, were of type IV(a) of IUPACs classification [23] which is typical of mesoporous materials. The presence of a H1 hysteresis loop indicates a narrow pore size distribution of uniform pores. The textural properties calculated from N_2_ adsorption/desorption isotherms for all samples studied are given in Table 1. SBA-15 and both Mn-containing SBA-15 supports show large specific surface areas (860–940 m^2^/g). As concerns the pore volume, it was higher for Mn(N)SBA-15 than for Mn(A)SBA-15. Interestingly, the pore volume was similar for Mn(N)SBA-15 and SBA-15 (pure silica without manganese), whereas the specific surface area and average pore size were higher for the sample containing Mn. In contrast, the specific surface area of Mn(A)SBA-15 was the same as that of SBA-15, whereas the average pore size was much lower. A comparison of pore size distribution in Mn(N)SBA-15 and Mn(A)SBA-15 (Figure 2) showed a significant difference between the shapes of PSD curves and maximum values (much higher for the first sample). Moreover, the secondary pore system in the range of 5–7 nm was more pronounced in Mn(A)SBA-15. These features, combined with a higher specific surface area of Mn(N)SBA-15, indicate a partial inclusion of manganese into the silica structure of Mn(N)SBA-15 as well as the location of a part of manganese species in the extra framework positions of Mn(A)SBA-15. As the unit cell parameter increased for both manganese-containing materials in comparison to the value for SBA-15 (a higher increase for Mn(N)SBA-15), one can conclude that also in Mn(A)SBA-15 a part of Mn species was included into silica framework. A similar observation has been reported in [24].

The values of specific surface area and pore volume significantly decrease for the samples modified with gold (surface area ~450–490 m^2^/g, pore volume 0.5–0.6 cm^3^/g). A similar behavior was also reported earlier for MCF [19] and SBA-15 mesoporous sieves [20], as a result of the functionalization of mesoporous solids with APTMS before modification with gold. The decrease in pore volume can be also a result of gold location in the pores of SBA-15, as shown in TEM images (Figure S2). However, gold introduction did not change the shape, maximum value, or width of the main peak in the PSD curve for Au-Mn(A)SBA-15, whereas it significantly diminished the broad peak coming from the system of secondary pores of smaller sizes (Figure 2). Therefore, taking into account that modification with gold led to the removal of a significant amount of manganese from Au-Mn(A)SBA-15, one can conclude that in Mn(A)SBA-15 a large part of manganese species was weakly bonded to the silica surface, thus easily removable during gold modification.

#### 2.1.2. Surface Characterization

**Figure 3:**
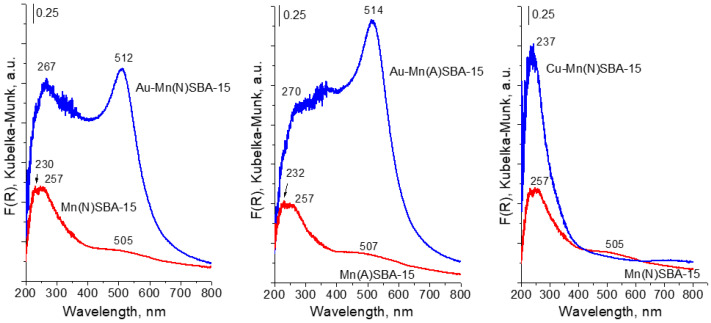
UV-vis spectra of MnSBA-15 materials before and after modification with gold or copper.

The state of metals (Mn, Au) in the SBA-15 materials was studied by XRD, UV-vis, and XPS techniques. The UV-vis spectra of Mn-containing supports are shown in Figure 3. For MnSBA-15 supports, three bands appeared in the UV-vis spectra: a broad band at ~500 nm and two less resolved bands at ~230 and 260 nm. The band at ~500 nm is attributed to d-d transition of Mn^2+^ in octahedral coordination [24,25]. The broad band in the range 200–350 nm with two maxima at 230 and 260 nm is due to charge transfer transition between O^2−^ and Mn^3+^ [25]. This indicates that both types of Mn species (Mn^2+^ and Mn^3+^) were present in Mn(N)SBA-15 and Mn(A)SBA-15. It should be noted that some authors assigned the band at ~270 nm to Mn^3+^ ions with tetrahedral coordination in the framework of mesoporous silica [18,24,26]. The broad band in the region between 200 nm and 350 nm can cover the bands assigned to the charge transfer transition between oxygen ions and manganese(III) cations in both extra-framework Mn(III) oxide species and framework Mn^3+^ in tetrahedral coordination with oxygen ions. Taking this into account, two oxidation states of Mn species can be distinguished in the supports: isolated framework or extra-framework Mn^3+^ species and Mn^2+^ species. The spectra of both MnSBA-15 samples are similar in shape. However, one cannot exclude the different Mn^2+^/Mn^3+^ ratios in both catalysts. As shown in [18], the intensities of bands at 270 and 500 nm, due to the different sensitivities, cannot be used to evaluate the ratios of Mn^2+^/Mn^3+^ species. The modification of manganese-containing supports with gold led to the appearance of an intensive band at ~500 nm in the UV-vis spectra, which is characteristic of SPR (surface plasmon resonance) of metallic gold [27,28]. It is important to note, that the band at ~260 nm coming from Mn^3+^ (isolated) species is still visible in the spectra of gold catalysts (Au-MnSBA-15) indicating the stability of this kind of species during the two-step modification with gold. In order to explain the stability of Mn^2+^ species (on the basis of the band at 500 nm overlapped by the SPR band in the UV-vis spectra of gold samples), an additional Cu-Mn(A)SBA-15 sample was prepared by exactly the same preparation procedure as Au-Mn(A)SBA-15 (a copper precursor instead of a gold one was applied) and subjected to UV-vis measurement. The UV-vis spectrum of Cu-Mn(N)SBA-15 does not show the band at ~500 nm (Figure 3), which proves the removal of Mn^2+^ species from Mn(N)SBA-15 as a result of modification with copper. It indicates that Mn^2+^ species are not stable. The results of ICP and UV-vis spectra provided the evidence that the removal of manganese species from Au-Mn(A)SBA-15 concerned mainly octahedral Mn^2+^ species.

Wide-angle XRD patterns (Figure 1B) also indicate the presence of metallic gold on the surface of both gold-modified MnSBA-15 catalysts. The reflections characteristic of metallic gold (at 2theta = 38.3° and 44.4°) [8] are well visible in the XRD patterns. However, no reflections corresponding to Mn oxides were observed because of a low loading of manganese in the samples.

**Figure 4:**
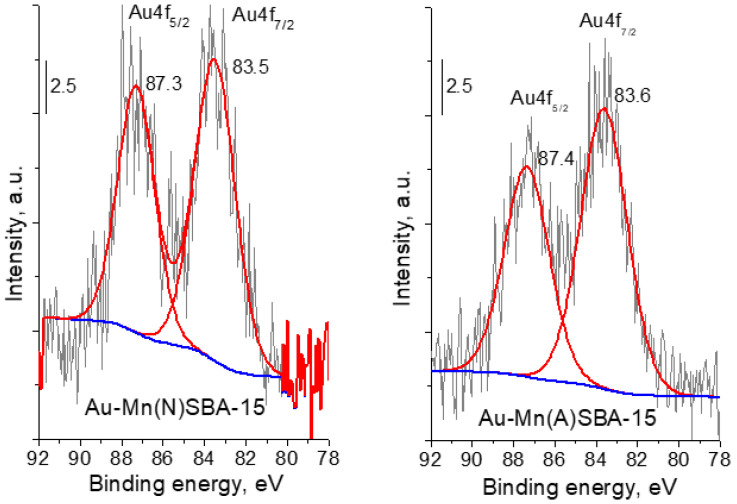
Au 4f region of XP spectra of gold-containing MnSBA-15 catalysts.

Another technique that confirmed the presence of metallic gold in SBA-15 catalysts was XPS spectroscopy. According to literature reports, the binding energy (BE) of metallic gold is 84.0 eV [29]. It is important to stress that for Au-SBA-15 and Au-MnSBA-15 the binding energy of Au 4f_7/2_ was lower, in the range of 83.6–83.4 eV (Figure 4 for Au-MnSBA-15 and [11] for Au-SBA-15—BE = 83.4 eV). The lower value of BE of gold than that of bulk Au^0^ metallic gold particles indicates the presence of negative charge on metallic gold particles loaded on the supports—(Au^0^)^δ−^ species [30,31]. The formation of such negatively charged metallic gold nanoparticles has been attributed in our previous works [11,19] to the presence of nucleophilic centers on the surface of silica support, which are able to donate electrons from the support to Au^0^ particles. The binding energy decreased in the following order: Au-Mn(A)SBA-15 > Au-Mn(N)SBA-15 > Au-SBA-15. However, the effect of Mn on the electronic properties of gold was not significant, but the tendency to increase in BE after Mn modification was notable.

**Figure 5:**
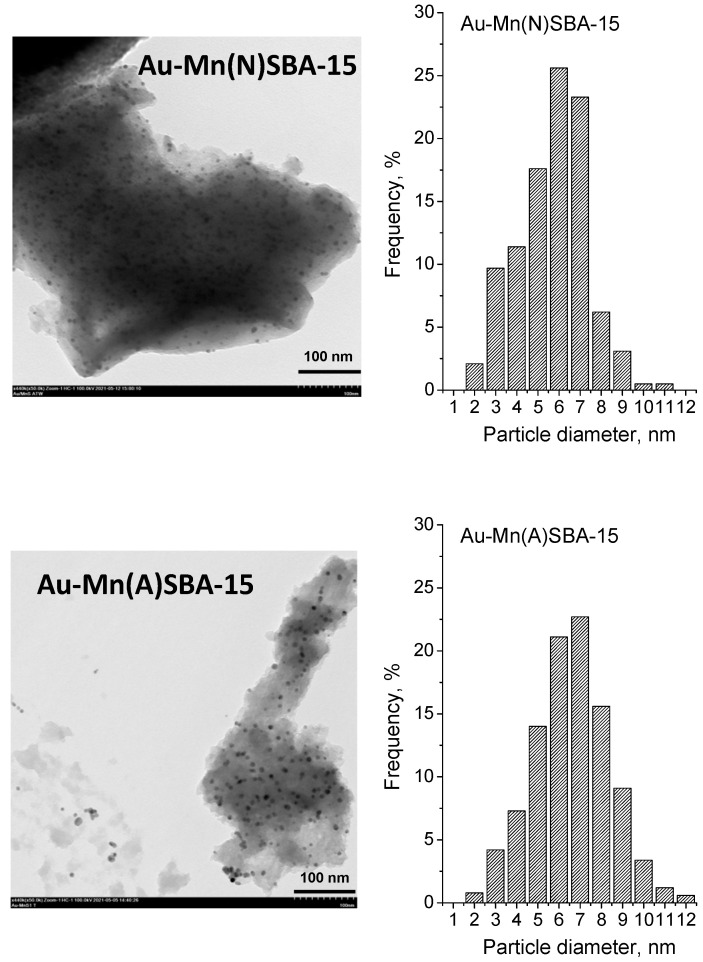
TEM images and gold particle size distribution histograms of Au-MnSBA-15 catalysts.

Transition electron microscopy (TEM) was used in order to get information about the particle size distribution of gold loaded on Au-SBA-15 (described in [11], Figure S3), Au-Mn(N)SBA-15, and Au-Mn(A)SBA-15 catalysts. The TEM images and particle size distribution diagrams are shown in Figure 5, whereas the average gold particle sizes are given in Table 1. The average diameter of gold particles (calculated from TEM images) was between 4.9 nm and 6.6 nm in all gold SBA-15 based catalysts and decreased in the following order: Au-Mn(A)SBA-15 (6.6 nm) > Au-Mn(N)SBA-15 (5.7 nm) > Au-SBA-15 (4.9 nm). It should be noted that the particle size distribution was different for the catalysts supported on SBA-15 and MnSBA-15 (Figure 5 and Figure S3 for Au-SBA-15). The majority (72%) of crystallites of gold particles on Au-SBA-15 showed diameters in a narrow range of 4–6 nm. Most gold particles on Au-Mn(N)SBA-15 and Au-Mn(A)-SBA-15 had diameters which varied in a wide range of 4–8 nm (~80%). A small fraction of larger particles (<8–11 nm) was also observed (4% and 14% for Au-Mn(N)SBA-15 and Au-Mn(A)-SBA-15, respectively). The results of elemental analysis indicated that the lower amount of APTMS was grafted on MnSBA-15 samples (2.4 mmol/g of N for Mn(N)SBA-15 and 2.3 mmol/g of N for Mn(A)SBA-15) than on SBA-15 (2.7 mmol/g of N). As a result, a lower number of NH_2_ groups on the MnSBA-15 surface interacted with the same amount of gold species and that is why larger Au particles were formed. This means that the preparation procedure of manganese-containing MnSBA-15 and the Mn precursor influenced the surface properties that determine the agglomeration of Au NPs.

### 2.2. Acidity

The acidity of SBA-15 catalysts modified with gold or manganese and gold was investigated by 2-propanol test reaction and FTIR study with pyridine adsorption as complementary techniques. The 2-propanol decomposition is a test reaction for acidic (Brønsted or Lewis) and basic properties, whereas the adsorption of pyridine allows a distinction between Brønsted and Lewis acid sites (BAS and LAS, respectively).

The infrared spectra (Figure S4) of both MnSBA-15 materials after pyridine adsorption at 423 K and after its desorption at increasing temperatures in the range 473–573 K, show the bands assigned to pyridine coordinatively chemisorbed on Lewis acid sites (LAS) [32,33], at ~1449 cm^−1^ and 1605 cm^−1^, coming from symmetric and antisymmetric vibrations in the chemisorbed probe molecules. Taking into account that the IR spectrum of mesoporous silica modified with gold after pyridine adsorption and temperature evacuation showed only the bands coming from physisorbed (1578 cm^−1^) and hydrogen bonded pyridine (1446 and 1597 cm^−1^) ([34], Figure S5), the bands at 1449 cm^−1^ and 1605 cm^−1^ visible in the spectra of the samples containing Mn should be assigned to pyridine adsorbed on acidic manganese species (LAS). The IR bands at ~1545 cm^−1^ and 1637 cm^−1^ typically assigned to Brønsted acid sites (BAS) were not detected. Moreover, the spectra after pyridine adsorption exhibited a pair of bands at 1446 cm^−1^ and at 1597 cm^−1^ assigned to hydrogen bonded pyridine. The band at 1490 cm^−1^ is typical of pyridine chemisorbed on both LAS and BAS, whereas the band at ~1576–1578 cm^−1^ comes from physisorbed pyridine.

As mentioned above, the IR bands at 1449 cm^−1^ and 1605 cm^−1^ come from pyridine coordinatively bonded to manganese cationic Lewis acid sites. The number of these sites was much higher on Mn(A)SBA-15 than on Mn(N)SBA-15 (see Table 2), although the amount of manganese in Mn(A)SBA-15 was lower (ICP results). This means that in Mn(N)SBA-15 a significant part of manganese species was not accessible to pyridine adsorption or did not play a role of LAS. Mn^3+^ introduced to the skeleton of silica does not act as LAS like aluminum in aluminosilicate SBA-15 if dehydroxylation is not performed. Moreover, a part of Mn^3+^ can be located in the walls of MnSBA-15 material and therefore is not accessible to the adsorbate. It is important to emphasize that the strength of LAS measured as the amount of pyridine desorbed at 573 K was much higher for Mn(A)SBA-15 than for Mn(N)SBA-15. The introduction of gold to Mn-containing materials resulted in a decrease in the number of LAS for Au-Mn(A)SBA-15 and almost did not change the number of LAS for Au-Mn(N)SBA-15, which resulted in a greater amount of LAS on Au-Mn(N)SBA-15 than on Au-Mn(A)SBA-15.

The results of 2-propanol decomposition reaction, shown in Table 3, clearly indicate the acidic character of both Mn-containing supports (>90% of selectivity to propene formed on acid sites [35]).

The conversion of 2-propanol obtained on Mn(A)SBA-15 was much higher than on Mn(N)SBA-15 (43 vs. 8%), which is in line with the LAS number identified by pyridine adsorption combined with the FTIR study (higher for Mn(A)SBA-15). The modification with gold changed the surface properties of the catalysts due to generation of basic centers (~50–60% of selectivity to acetone, formed on basic sites [35]). Negatively charged metallic gold particles identified by XPS measurements are the basic centers. The activity and selectivity to propene for Au-Mn(A)SBA-15 decreased in comparison to that noted for Mn(N)SBA-15. The reason is the removal of the unstable manganese extra framework species (LAS centers) during the modification with gold, proved by ICP and UV-vis results and confirmed by pyridine adsorption study. It proves the domination of the extra framework manganese oxides (localized in pores—see textural parameters), playing the role of LAS with low stability (easily removed), in Mn(A)SBA-15. It demonstrates that Mn(N)SBA-15 contains mainly stable isolated Mn^3+^ species which are included into silica matrix and do not act as LAS. On the other hand, the activity of Au-Mn(N)SBA-15 increased as a result of gold introduction because of generation of additional basic centers (negatively charged gold nanoparticles). They are less important in 2-propanol decomposition on Au-Mn(A)SBA-15 rich in LAS.

### 2.3. Glucose Oxidation

#### 2.3.1. Interaction of Catalysts with Glucose and H_2_O_2_—ATR-FTIR and UV-Vis Studies

The adsorption/interaction of reagents (glucose and hydrogen peroxide) on the surface of selected catalysts combined with ATR-FTIR and UV-vis spectroscopy study were performed in order to investigate which kind of active centers take part in such interaction and in which form the substrates can be chemisorbed.

**Figure 6:**
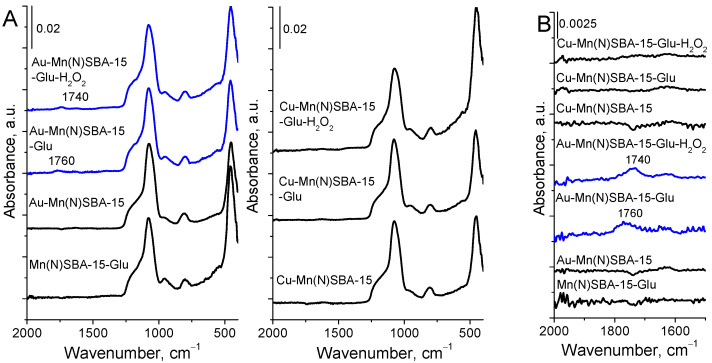
ATR-FTIR spectra of selected MnSBA-15 materials before and after glucose (Glu) or glucose and H_2_O_2_ solution treatment and drying at 353 K. (**A**) The range of 2000–400 cm^−1^; (**B**) the range of 2000–1500 cm^−1^.

The interaction of glucose with the surface of Mn(N)SBA-15, Au-Mn(N)SBA-15, and Cu-Mn(N)SBA-15 materials was estimated on the basis of ATR-FTIR spectra of the catalysts after adsorption of glucose solution and drying at 353 K (Figure 6).

In all the spectra of the catalysts, before and after treatment with glucose, the bands in the region of 1500–400 cm^−1^ corresponding to the Si-O-Si antisymmetric and symmetric stretching vibrations in silica, are well visible. Moreover, in the spectra of Au-Mn(N)SBA-15 after adsorption of glucose or glucose with H_2_O_2_, the bands in the C=O vibration region (1800–1600 cm^−1^) appeared (at 1760 cm^−1^ and 1740 cm^−1^, respectively). These bands were previously assigned to the chemisorption of glucose with the participation of protons and water [12]. The glucose–water–proton complex contains carboxyl groups whose C=O vibrations are manifested in the IR spectrum as these two bands. A similar complex with the participation of LAS, not BAS (protons), via formation of coordinative bond between LAS and oxygen from hydroxyl group at C6 or carbonyl oxygen (at carbon atom C1) in glucose, as proposed in [11], should also give rise to the appearance of an IR band coming from C=O vibrations. However, glucose adsorption on Mn(N)SBA-15 containing an even higher amount of LAS than the gold-containing material did not lead to the appearance of the above-mentioned band. Moreover, in spite of a much higher number of LAS in Cu-Mn(N)SBA-15 (LAS coming from both manganese and copper cationic species—Table 2), the band from the above-mentioned complex was not observed in the spectrum after glucose adsorption and interaction with H_2_O_2_ (Figure 6B). Thus, one can conclude that glucose is not chemisorbed on LAS coming from the support manganese species as well as from copper Lewis acid sites. Therefore, it is clear that both adsorption and oxidation of glucose occur with the participation of gold NPs centers. The other possibility which should be considered is the assignment of the mentioned bands to C=O stretching vibrations in the reaction product, that is gluconic acid [12]. The shift of this band to a lower wavenumber (from 1760 cm^−1^ to 1740 cm^−1^) after hydrogen peroxide admission to the sample with adsorbed glucose can be explained as a result of the oxidation of glucose to gluconic acid, and the band at 1740 cm^−1^ can be assigned to C=O vibration in carboxylic group of acid.

**Figure 7:**
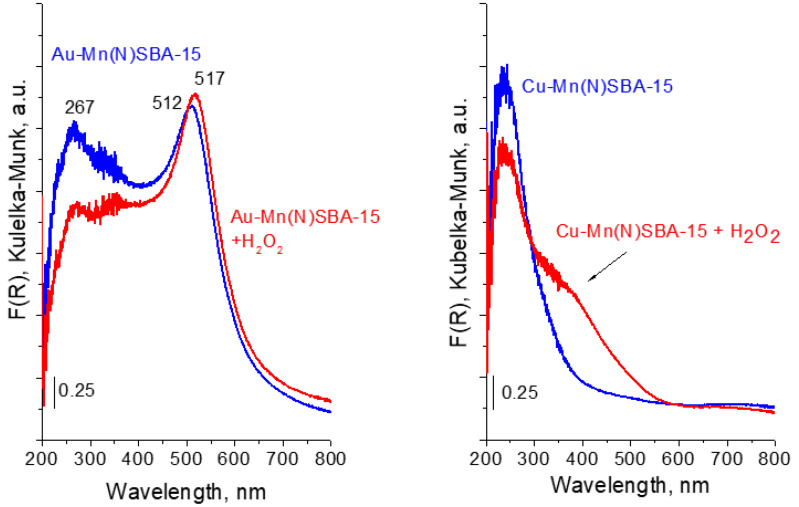
UV-vis spectra of Au-Mn(N)SBA-15 and Cu-Mn(N)SBA-15 before and after treatment with H_2_O_2_.

To study the interaction of the catalyst surfaces with H_2_O_2_, the Au-Mn(N)SBA-15 and Cu-Mn(N)SBA-15 samples were contacted with hydrogen peroxide. The changes in the UV-vis spectra were observed for Cu-Mn(N)SBA-15 (Figure 7). The spectrum showed an increase in the absorption intensity in the range 300–450 nm, indicating the formation of peroxo/superoxo species [36,37]. This means that, on copper-containing sample, hydrogen peroxide is decomposed to HOO^•^ radicals and/or peroxo species [36,37,38]. 

For Au-Mn(N)SBA-15, the region characteristic of peroxo/superoxo species is overlapped by a strong band characteristic of SPR of AuNPs (Figure 7). Thus, it is not possible to estimate the possibility of superoxo radicals formation. A small shift of the maximum of the SPR band (from 512 nm to 517 nm) suggests the interaction of Au NPs with hydrogen peroxide. Interestingly, during the interaction of hydrogen peroxide with gold catalysts, gas evolution was observed. According to literature, e.g., [16], the adsorption of H_2_O_2_ on Au-surface-active species is accompanied by electron transfer from Au to H_2_O_2_ and, next, decomposition of hydrogen peroxide occurs, according to the redox mechanism. In this mechanism the adsorbed H_2_O_2_ accepts electron from Au NPs to decompose to OH^−^ ions and HO^•^ radicals, OH^−^ ions reacts with free H_2_O_2_ to form HO_2_^−^ ions and water, HO_2_^−^ ions interact with adsorbed HO^•^ to molecular oxygen and water.

#### 2.3.2. Activity and Selectivity

The activity of gold-containing MnSBA-15 catalysts was studied in microwave assisted base-free oxidation of glucose. Hydrogen peroxide was used as oxidizing agent. The activities of MnSBA-15 supports as well as Au-Mn(N)SBA-15 and Au-Mn(A)SBA-15 catalysts are shown in Table 4 and are compared with those of Au-SBA-15 and Cu-Mn(N)SBA-15. The reaction was performed at 383 K for 10 min. Under these conditions, only slight glucose conversion was observed for Mn(N)SBA-15 and Mn(A)SBA-15 supports. The introduction of gold to the catalysts generated the activity. The activity expressed as the conversion of glucose was only slightly lower for Au-SBA-15 than Au-Mn(N)SBA-15 (64% and 66%, respectively), whereas that expressed in terms of TOF values was considerably higher for Au-Mn(N)SBA-15 than for Au-SBA-15. The smaller size of gold particles on Au-SBA-15 surface (a higher number of accessible gold active sites at a similar gold loading) effected a lower activity of a single gold accessible species and, therefore, TOF was lower for this catalyst. However, the relations between the glucose conversion and the size of AuNPs as well as between the TOF values and the size of AuNPs were not linear. In [13] it has been indicated that the electron density on the surface of Au NPs is responsible for the electron transfer in the first step of the redox mechanism proposed for the decomposition of hydrogen peroxide to molecular oxygen and water. In the case of gold catalysts applied in this work, the electron density on Au NPs, estimated from binding energy shown in XPS spectra (Figure 4), was similar (only slightly different).

The difference in activity of Au-Mn(A)SBA-15 (the lower glucose conversion and TOF) and Au-Mn(N)SBA-15 (the higher conversion of glucose and TOF) could be considered in relation to the Au NPs’ size (greater for Au-Mn(A)SBA-15), the number of LAS coming from manganese species (lower for Au-Mn(A)SBA-15), and the pore size (lower for Au-Mn(A)SBA-15). Although Omri et al. [10] have shown that, in the basic conditions in MW assisted oxidation of glucose, no difference in the activity of Au/Al_2_O_3_ catalyst with 2.4-nm-sized and 7.4-nm-sized gold nanoparticles was noted, in our study under base-free conditions, the maximum activity was observed for the sample with 5.7 nm average size of Au NPs (Au-Mn(N)SBA-15). The nonlinear relationship between the Au NPs’ size and the activity in oxidation with H_2_O_2_ has been observed also by the other authors for the oxidation performed on gold catalysts with H_2_O_2_, e.g., [16]. In the mentioned paper the authors used Au/TiO_2_ catalysts and found out that the catalytic activity for the decomposition of H_2_O_2_ strongly depended on the Au NPs’ size, with a maximum for the size of 4.6 nm, as a result of the size-dependences of the number of active sites and the Fermi energy (E_F_) of Au NPs. The smaller the Au nanoparticles, the higher the number of low-coordinated active sites responsible for H_2_O_2_ chemisorption, whereas the E_F_ decreases with lowering of Au NPs’ size. Thus, on the one hand, the lowering of gold particle size decreasing Fermi energy would depress electron transfer from Au NPs to H_2_O_2_, but on the other hand, small gold particles provide more active sites for H_2_O_2_ adsorption. The value of E_F_ of gold particles depends on the type of support. This is the reason why in various papers one can find different optimal sizes of AuNPs for the most effective oxidation with hydrogen peroxide. In this paper for gold catalysts loaded on SBA-15 and MnSBA-15, the optimal Au NPs’ average size appeared to be 5.7 nm. It is important to add that the negative charge of gold nanoparticles is also determined by the chemical composition of the support. The results presented in this paper show that for similar negative charge of Au NPs, their size, which determines both the number of active gold atoms and Fermi energy, is an important factor affecting the activity in base-free microwave assisted glucose oxidation.

To check if other parameters have an impact on the activity of the catalysts studied, we considered the role of LAS and pore size of the catalysts. All three gold catalysts used in this work exhibited different acidity and their acidities could be involved in the reaction pathway that includes chemisorption of glucose. Au-SBA-15 did not exhibit acidity, but it was highly active in glucose oxidation. Moreover, it was found (Section 2.3.1) that glucose was not chemisorbed on manganese LAS in Mn-containing supports, but on Au NPs. Thus, it can be concluded that there is the optimum size of gold particles (average—5.7 nm) for adsorption of glucose and decomposition of H_2_O_2_ under MW, smaller than that proposed for oxidation of glucose with oxygen on gold-containing zeolites [12,13] under atmospheric pressure (15–30 nm). In order to check whether molecular oxygen can oxidize glucose in the presence of the gold catalysts based on MnSBA-15 used in this work, the reaction was performed in the pressure batch reactor under molecular oxygen pressure of 0.5 MPa (20 mL of 0.2 M glucose solution, glucose/Au (molar ratio) = 1970/1). The activity and selectivity of this reaction after 120 min are presented in Table S1. The copper-containing catalyst was inactive, whereas the activity of the gold catalyst was very high (86% of glucose conversion) with 99.5% selectivity to gluconic acid. On the other hand, Cu-Mn(N)SBA-15 showed almost the same activity as Au-Mn(N)SBA-15 (Table 4) in the MW-assisted oxidation of glucose with hydrogen peroxide. The important difference between both catalysts is the selectivity of the reaction. The dominant product of glucose oxidation, gluconic acid, was formed over the gold-containing sample, indicating superior selectivity of this catalyst to the target product. In contrast, the Cu-Mn(N)SBA-15 sample was not selective and many products were formed, suggesting completely different pathways of glucose oxidation on gold and copper catalysts. As the UV-vis study of the copper catalyst treated with H_2_O_2_ proved the formation of superoxide species, the radical mechanism of glucose oxidation was proposed for this catalyst. It was not the case for Au-MnSBA-15, in whose presence a decomposition of H_2_O_2_ to oxygen was observed. The high activity of gold catalyst in glucose oxidation with oxygen (Table S1) confirmed that molecular oxygen from decomposition of H_2_O_2_ can be effective in glucose oxidation on gold particles.

**Figure 8:**
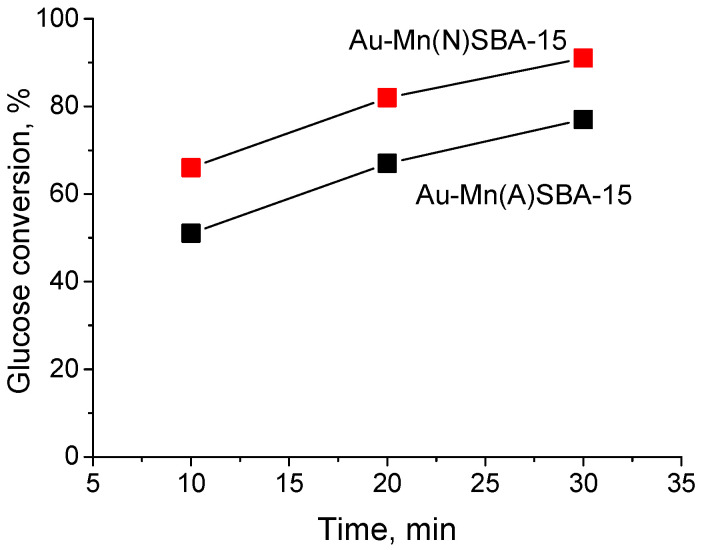
Effect of the reaction time on the activity of Au-Mn(N)SBA-15 and Au-Mn(N)SBA-15 in MW-assisted base-free glucose oxidation with H_2_O_2_; reaction conditions: glucose/Au (molar ratio) = 1970/1, 8 mL of 0.2 M glucose solution, T = 383 K, 2.2 equiv. H_2_O_2_.

The impact of pore size on the reaction rate was investigated for Au-Mn(N)SBA-15 (mean pore size 10.6 nm) and Au-Mn(A)SBA-15 (mean pore size 8.3 nm) (Figure 8). The reaction was performed for 10, 20, and 30 min. For both samples, the increase in glucose conversion with increasing reaction time was observed. Although the difference in pore sizes between the two samples was significant, the curves illustrating the increase in the activity with the reaction time are parallel. This indicates that diffusion of reagents in pores does not limit the reaction rate, and means that the size of mesopores is not a factor which affected the activity of catalysts studied in this work.

The effect of temperature on the glucose oxidation was studied further for the best catalyst—Au-Mn(N)SBA-15 (Table 5). The reaction was performed in the range 383–413 K (every 20 K). The observed increase in the glucose conversion with the temperature proved that a reaction takes place in the kinetic regime. Simultaneously, a decrease in the selectivity to gluconic acid was noted with increasing reaction temperature. The formation of glucuronic acid as a side product was observed for the reaction performed at 403 and 413 K. The lowering of selectivity to gluconic acid with increasing reaction temperature was observed also for Au/Al_2_O_3_ studied in glucose conversion with H_2_O_2_ [9,39].

**Figure 9:**
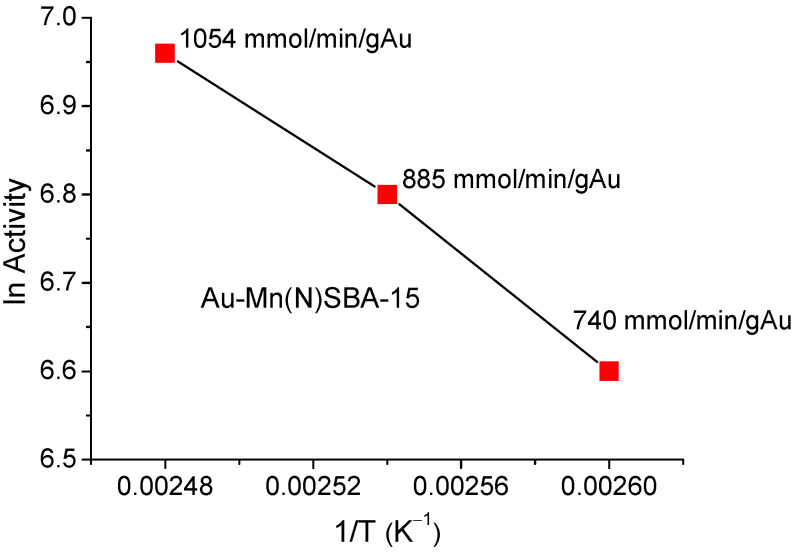
Arrhenius plot for the determination of the apparent activation energy.

Based on the activity values shown in Table 5 for the temperature range 383–403 K (413 K was not taken into account because of a higher participation of side reaction activity), an Arrhenius plot was drawn (Figure 9) and the apparent activation energy was calculated to 25 kJ/mol. This energy was much lower than that calculated in [39] for the reaction performed on Au/Al_2_O_3_ in the conventional system with the use of base in the reaction media and H_2_O_2_ as oxidant (48 kJ/mol). It is in line with many observations, e.g., [40,41], that microwave irradiation decreased the value of the activation energy of the reaction relative to that needed in conventional conditions.

In order to compare the catalytic activity of Au-MnSBA-15 with those of the other gold catalysts studied earlier in microwave-assisted base-free glucose oxidation with hydrogen peroxide, the activity of gold catalysts described in the literature is summarized in Table 6.

Entries 1–6 concern the reaction performed on Au-catalysts containing a similar gold loading (~2% wt. %). As for entries 5 and 6, the data from literature were recalculated according to TOF formula used in this work to be able to make a comparison. Entry 7 presents the results for the reaction carried out in the presence of NaOH in the reaction medium and for the catalyst with ~5 times lower gold loading. Therefore, the TOF number was not recalculated.

It is clear that Au-Mn(N)SBA-15 is characterized by the highest TOF value from among the compared catalysts (entries 1–6), even though the Glu/Au molar ratio applied in [9] was lower and the reaction temperature was higher (entries 5 and 6) than those used in this work. Moreover, it is noteworthy that Au-MnSBA-15 materials show almost 100% selectivity to gluconic acid. It should be admitted that the activities of gold catalysts in base-free reaction conditions are typically lower than when the reaction is performed in the presence of NaOH (entry 7). However, if basic conditions are used, gluconate salt is finally obtained instead of pure gluconic acid, and some basic waste is formed. The use of basic media also leads to the glucose to fructose isomerization which is favored at high pH [42]. Because of the inconveniences coming from the use of basic media, base-free selective oxidation of glucose on gold catalyst is desirable even if lower conversion of glucose is obtained.

## 3. Materials and Methods

### 3.1. Materials and Chemicals

The chemicals used in this work were: Pluronic P123 (Poly(ethylene glycol)-block-Poly(ethylene glycol)-block-Poly(ethylene glycol)-block) copolymer (Sigma-Aldrich, Saint Louis, MO, USA), tetraethyl orthosilicate (TEOS) (Sigma-Aldrich, Saint Louis, MO, USA, 98%), hydrochloric acid (35–38% HCl, POCH S.A. Gliwice, Poland), manganese(II) nitrate hydrate (Sigma-Aldrich, Saint Louis, MO, USA, 99.99%), manganese(II) acetate (Sigma-Aldrich, Saint Louis, MO, USA, 98%), chloroauric acid (HAuCl_4_∙*x*H_2_O, Sigma Aldrich, Saint Louis, MO, USA, 99.995%), copper(II) nitrate (Sigma-Aldrich, Saint Louis, MO, USA, 99.99%), sodium borohydride (Sigma Aldrich, Saint Louis, MO, USA, >98%), (3-aminopropyl)trimethoxysilane (APTMS, Sigma Aldrich, St. Louis, MO, USA, 97%), 2-propanol (p.a., StanLab, Lublin, Poland), pyridine (C_5_H_5_N, Sigma Aldrich, 99.8%, glucose (Sigma Aldrich, Saint Louis, MO, USA, ≥99.5%), hydrogen peroxide (30 wt. %, StanLab, Lublin, Poland) and deionized water.

### 3.2. Preparation of Catalysts

MnSBA-15 mesoporous molecular sieves were synthesized using two manganese precursors (manganese(II) nitrate—denoted as (N) and manganese(II) acetate—denoted as (A)) according to procedures described in [24,25] with some modifications.

#### 3.2.1. Synthesis of Mn(N)SBA-15

Pluronic P123 (4 g) was dissolved in 170 g mL of distilled water. Then the pH was adjusted to 3 by adding HCl (35–38%) (solution A). Tetraethyl orthosilicate (TEOS) (22 g) was mixed with manganese(II) acetate (Si/Mn = 9) in a separate container (solution B). Then solution B was added dropwise to solution A and the combined mixture was vigorously stirred at 313 K for 24 h. The resulting mixture was heated at 373 K for 24 h. The obtained solid was precipitated, washed with distilled water, and dried at 373 K. In the last step, the template was removed by calcination at 813 K for 8 h at a heating rate of 1 K min^−1^. The prepared sample was denoted as Mn(N)SBA-15 (where N denotes manganese(II) nitrate used as Mn precursor).

#### 3.2.2. Synthesis of Mn(A)SBA-15 

Pluronic P123 (4 g) was dissolved in 150 g mL of distilled water. Then the pH was adjusted to 3 by adding HCl (35–38%) (solution A). Tetraethyl orthosilicate (TEOS) (9 g) was mixed with manganese(II) nitrate (Si/Mn = 4) and dissolved in 20 mL of water in a separate container (solution B). Then solution B was added dropwise to solution A and the combined mixture was vigorously stirred at 313 K for 24 h. The resulting mixture was heated at 373 K for 24 h. The obtained solid was precipitated, washed with distilled water, and dried at 373 K. In the last step, the template was removed by calcination at 813 K for 8 h at a heating rate of 1 K min^−1^. The prepared sample was denoted as Mn(A)SBA-15 (where A denotes manganese(II) acetate used as Mn precursor).

#### 3.2.3. Modification of MnSBA-15 Supports with Gold

The modification of MnSBA-15 supports with gold was performed according to the two-step procedure proposed by Mou [21]. First, Mn(N)SBA-15 and Mn(A)SBA-15 were functionalized with APTMS ((3-aminopropyl)-trimethoxysilane) [10,19]. Next, the obtained functionalized MnSBA-15 was stirred for 1 h at room temperature with aqueous solution of chloroauric acid (HAuCl_4_∙3H_2_O) as a metal precursor (2.2 wt. % of Au as assumed). After filtration and washing, the precipitation was stirred for 20 min with 40 mL of 0.1M NaBH_4_ solution used as a reducing agent. The solid product was recovered by filtration and washing with water. The final materials were obtained by drying at 373 K and calcination at 773 K for 4 h.

#### 3.2.4. Reference Material—Modification of Mn(N)SBA-15 Support with Copper

For the preparation of Cu-Mn(N)SBA-15, the same two-step procedure as that for gold catalysts (Section 3.2.3) was applied, but in the second step, Cu was deposited on Mn(N)SBA-15 support using Cu(NO_3_)_2_ as the precursor (2.2 wt. % of Cu as assumed).

### 3.3. Catalysts Characterization

The actual gold content in the catalysts was determined with the use of inductively coupled plasma optical emission spectrometry with an ICP-OES SPECTRO BLUE TI spectrometer (Kleve, Germany). The gold phase was extracted from the sample by means of aqua regia in a microwave oven.

The XRD patterns were recorded on a D8 Advance diffractometer (Bruker) (Billerica, MA, USA) using Cu Kα radiation (λ = 0.154 nm), with a step size of 0.05° in the small-angle range (1°–6°) and with a step size of 0.2° in the wide-angle range (20°–60°).

The nitrogen adsorption−desorption isotherms of all catalysts were measured at 77 K using a Micromeritics ASAP 2020 Physisorption Analyzer (Norcross, GA, USA). Before measurements, the samples were degassed at 573 K for 8 h. The surface area of the materials obtained was calculated with the use of the Brunauer−Emmett−Teller (BET) method. The pore volume and diameter were determined by DFT method appropriate for mesoporous silicas with cylindrical pore system using MicroActive software (Version 4.0) from Micromeritics.

Elemental analyses of the samples after grafting with APTMS were carried out on a FLASH 2000 Elemental Analyser.

For transmission electron microscopy (TEM) measurements, the suspensions of the powders in absolute ethanol were deposited on a copper grid covered with a carbon film and transferred to a Hitachi HT7700 electron microscope (Hitachi High-Tech, Tokyo, Japan) operating at an accelerating voltage of 100 kV.

UV-vis spectra were recorded using a Varian Carey 300 Scan UV-visible spectrophotometer (Candela, Warszawa, Poland). Powder samples were placed in a cell equipped with a quartz window. The spectra were recorded in the range from 800 to 190 nm. Spectralon was used as the reference material.

X-ray photoelectron spectroscopy (XPS) was performed on an ultra-high vacuum photoelectron spectrometer based on a PHOIBOS 150 NAP analyzer (Specs, Berlin, Germany). The analysis chamber was operated under vacuum with a pressure close to 5 × 10^−9^ mbar and the sample was irradiated with a monochromatic Al _Kα_ (1486.6 eV) radiation (15 kV; 10 mA). The spectra were recorded with a flood gun acting as neutralizer. Binding energies were referenced to the C1s peak from carbon, to the assumed value of 284.8 eV.

The attenuated total reflectance Fourier transform infrared (ATR-FTIR) spectra were measured on a Bruker Vertex 70 spectrometer (Billerica, MA, USA) equipped with an ATR attachment with a diamond crystal plate. The catalysts in portions of 15 mg were treated with 0.5 mL of 0.2 M glucose solution and heated at 353 K, then placed over the diamond crystal, and force was applied to the sample by rotation of the pressure clamp to its click-stop release. The spectra were recorded in the range from 4000 to 400 cm^−1^.

### 3.4. Acidity Measurements

Pyridine (Py) adsorption was measured with a Bruker Invenio S spectrometer (Billerica, MA, USA) at room temperature in the range from 4000 to 400 cm^−1^. The samples pressed at ~30 bar into thin wafers with density of ~8–12 mg∙cm^−2^ were placed inside a vacuum cell. Before measurements, the catalysts were evacuated at 623 K for 2 h. Then, Py was introduced into the cell at 423 K and, after the saturation, the excess of pyridine was removed by degassing in vacuum at 423 K for 5 min. Next, the samples were degassed at 423, 473, 523, and 573 K under vacuum for 30 min at each temperature. The spectrum without adsorbed pyridine (after activation) was subtracted from all recorded spectra. The number of Lewis acidic sites were calculated assuming the extinction coefficient ε for the band at 1450 cm^−1^ = 2.22 µmol^−1^ cm [31].

The 2-propanol dehydration and dehydrogenation test reaction was performed using a microcatalytic pulse reactor inserted between the sample inlet and the column of a SRI 310C gas chromatograph (Torrance, Kalifornia, USA). A portion of 0.05 g of the granulated catalyst was activated at 623 K for 2 h under nitrogen flow (40 cm^3^ min^−1^). The 2-propanol conversion was studied at various temperatures (423–573 K) using 3 μL pulses of alcohol under nitrogen flow (40 cm^3^ min^−1^). The substrate was vaporized before being passed through the catalyst bed with the flow of nitrogen carrier gas. The reaction mixture was separated on a 2 m column filled with Carbowax 400 loaded on Chromosorb W (80–100 mesh) and detected by FID.

### 3.5. Base-Free Oxidation of Glucose

#### 3.5.1. Reaction Conditions

The oxidation reactions with hydrogen peroxide as an oxidant were performed in a microwave synthesis platform MicroSYNTH (from Milestone, Sorisole, Italy) equipped with a contactless temperature infrared controller. In a typical experiment, 8 mL of 0.2 M aqueous glucose solution was used. Firstly, 0.2883 g of glucose in 7.64 mL of deionized water was introduced into a 10 mL glass tube. Then, an appropriate amount of a given catalyst (glucose/Au molar ratio: 1970/1) was added. Prior to testing in the reaction, all catalysts were dried in the oven at 423 K for 12 h to remove adsorbed water. Finally, 0.36 mL of a hydrogen peroxide solution was introduced. The reaction was performed without base addition. The H_2_O_2_ to glucose molar ratio was equal to 2.2. The reactions were conducted for 10 min at 383 K (stirring rate: 80% of the maximum setting). Afterwards, the reaction mixtures were withdrawn using a syringe, filtered through Millipore filter (0.2 µm, PTFE), and analyzed by ultrahigh-performance liquid chromatography on a UPLC Acquity Arc Waters instrument (Section 3.5.2). In order to study the effect of reaction time and temperature, additional reactions were performed over a selected catalyst for 20 and 30 min at 383 K or for 10 min at 393, 403, and 413 K. The other conditions remained unchanged.

#### 3.5.2. Analysis of Reactant and Products

Quantitative analyses of the reaction mixtures were performed using an ultrahigh-performance liquid chromatograph UPLC Acquity Arc Waters (Milford, MA, USA) and the products of the reaction were analyzed by two detectors, 2414 RI and 2998 PDA. The reactant and the products were separated on Shodex sugar column SH1011 heated at 303 K. The eluent was an aqua solution of H_2_SO_4_ (0.005 M), and its flow rate was set at 0.6 mL min^−1^. The samples to be analyzed were collected at the end of the reaction: 1 mL of the reactant/products solution was diluted in 25 mL of deionized water.

## 4. Conclusions

The results presented in this paper confirmed that in microwave assisted base-free oxidation of glucose with hydrogen peroxide on gold catalysts, the crucial step in selective formation of gluconic acid is decomposition of H_2_O_2_ to molecular oxygen and water. The decomposition of hydrogen peroxide to hydrosuperoxo radicals on copper-containing material led to unselective glucose oxidation. It has been indicated that not only negative charge accumulated on the Au NPs (expressed by BE estimated from XPS spectra) determines the rate of electron transfer from gold to H_2_O_2_ in the first step (limiting the reaction rate) of redox decomposition of hydrogen peroxide to oxygen, but also the amount of accessible gold atoms (greater for smaller size Au nanoparticles) and Fermi energy (E_F_) of Au NPs (lower for smaller size of Au nanoparticles). The lower the E_F_ value, the lower the rate of electron transfer from Au NPs to H_2_O_2_. Thus, to achieve effective activity in the reaction studied, for similar negative charges accumulated on Au NPs as in this work, a compromise between the number of active gold species and the level of E_F_ has to be reached. For the catalysts studied in this work it was achieved for Au NPs of 5.7 nm in average diameter. It should be added that Au NPs’ size was affected by the presence of Mn in SBA-15.

The introduction of manganese into SBA-15 support for gold also had an impact on the number of Lewis acid sites (LAS) and the pore size. Both parameters depended on the Mn precursor used and the synthesis protocol. It has been indicated that LAS coming from the support (manganese species) do not chemisorb glucose and, therefore, do not participate directly in the reaction pathway. Glucose is chemisorbed on Au NPs, i.e., on the same active centers on which decomposition of hydrogen peroxide to molecular oxygen occurs via the redox mechanism. It has been also demonstrated that the transport of reagents in mesopores did not limit the reaction rate. Moreover, as follows from the activity dependence on the reaction temperature obtained for the best catalyst, Au-Mn(N)SBA-15, the activation energy was 25 kJ/mol, which is much lower than for the reaction performed under conventional conditions without microwave heating.

## Figures and Tables

**Table 1:** The chemical composition, mean Au NP sizes and textural properties of catalysts.

Entry	Catalyst	wt. % Au or Cu ^a^	wt. % Mn ^a^	Au NPs Size ^b^, nm	Unit Cell Parameter, nm	BET Surface Area, m^2^ g^−1^	Total Pore Volume, DFT, cm^3^ g^−1^	Average Pore Diameter, DFT, nm
1.	SBA-15 ^c^	–	–	–	11.0	860	1.05	10.7
2.	Au-SBA-15 ^c^	2.1	–	4.9 ± 1.4	-	474	0.68	9.6
3.	Mn(N)SBA-15	–	0.50	–	11.6	936	1.00	11.2
4.	Au-Mn(N)SBA-15	2.2	0.46	5.7 ± 1.6	-	492	0.65	10.6
5.	Mn(A)SBA-15	–	0.20	–	11.4	862	0.75	8.3
6.	Au-Mn(A)SBA-15	2.1	0.13	6.6 ± 1.8	-	478	0.51	8.3
7.	Cu-Mn(N)SBA-15	2.2	0.48	-	-	661	0.64	10.6

^a^ Gold, copper, and manganese loading determined by ICP-OES. ^b^ The mean gold particle sizes calculated from TEM images. ^c^ on the base of [11].

**Table 2:** The number of Lewis acid sites (LAS) occupied by pyridine after adsorption at 423 K and desorption at different temperatures (calculated by the use of extinction coefficient from [32], ε_1450_ = 2.22 µmol^−1^ cm).

Entry	Catalyst	Evacuation Temp., K	Number of LAS, μmol g^−^^1^	Pyridine Desorbed at 573 K from LAS, % ^a^
1.	Mn(N)SBA-15	523	8.8	
		573	4.6	48
2.	Au-Mn(N)SBA-15	523	7.9	
		573	4.6	42
3.	Mn(A)SBA-15	523	9.5	
		573	6.8	28
4.	Au-Mn(A)SBA-15	523	5.6	
		573	3.8	32
5.	Cu-Mn(N)SBA-15	523	40.6	
		573	32.8	19

^a^ Related to the amount of pyridine chemisorbed after evacuation at 523 K.

**Table 3:** Results of 2-propanol decomposition at 573 K.

Entry	Catalyst	2-Propanol Conv., %	Selectivity, %		
			Propene	Acetone	Ether
1.	Au-SBA-15 ^a^	1	traces	traces	traces
2.	Mn(N)SBA-15	8	91	6	3
3.	Au-Mn(N)SBA-15	16	48	50	2
4.	Mn(A)SBA-15	43	100	0	0
5.	Au-Mn(A)SBA-15	19	33	66	1

^a^ on the basis of [11].

**Table 4:** Results of microwave-assisted oxidation of glucose with hydrogen peroxide over SBA-15 catalysts.

Entry	Catalyst	Glucose Conv. ^a^, %	Selectivity, %		TOF ^b^, h^−1^
			A	B	
1.	Mn(N)SBA-15	~1	traces	0	-
2.	Mn(A)SBA-15	3	traces	0	-
3.	Au-SBA-15	64	~100	traces	145,144
4.	Au-Mn(N)SBA-15	66	~100	traces	173,034
5.	Au-Mn(A)SBA-15	51	~100	traces	155,790
6.	Cu-Mn(N)SBA-15	67	ni ^c^	ni ^c^	-

^a^ Reaction conditions: glucose/Au (molar ratio) = 1970/1, 8 mL of 0.2 M glucose solution, T = 383 K, 2.2 equiv. H_2_O_2_, time = 10 min.; A—gluconic acid, B—glucuronic acid. ^b^ The number of moles of gold atoms localized on the external surface of spherical Au particles were considered in TOF calculation: (number of moles of glucose converted after 10 min) × (number of moles of gold atoms localized on the external surface of the Au NPs in a given mass of the catalyst)^−1^ × h^−1^; based on Au NPs’ size calculated from TEM. ^c^ ni—not identified.

**Table 5:** Results of microwave-assisted oxidation of glucose with hydrogen peroxide over Au-Mn(N)SBA-15 catalyst—the effect of the reaction temperature.

Entry	Catalyst	Temp., K	Glucose Conv. ^a^, %	Selectivity, %		TOF ^b^, h^−1^
				A	B	
1.	Au-Mn(N)SBA-15	383	66	~100	traces	173,034
2.	Au-Mn(N)SBA-15	393	79	~100	traces	207,116
3.	Au-Mn(N)SBA-15	403	94	99.7	0.3	246,442
4.	Au-Mn(N)SBA-15	413	97	99.0	1.0	254,307

^a^ Reaction conditions: glucose/Au (molar ratio) = 1970/1, 8 mL of 0.2 M glucose solution, 2.2 equiv. H_2_O_2_, time = 10 min.; A—gluconic acid, B—glucuronic acid. ^b^ The number of moles of gold atoms localized on the external surface of spherical Au particles were considered in TOF calculation: (number of moles of glucose converted after 10 min) × (number of moles of gold atoms localized on the external surface of the Au NPs in a given mass of the catalyst)^−1^ × h^−1^; based on Au NPs’ size calculated from TEM.

**Table 6:** Comparison of activity of different gold catalysts in microwave-assisted glucose oxidation with hydrogen peroxide.

Entry	Catalyst	wt. % Au	Au NPs Size, nm	Reaction Conditions	TOF, h^−1^	Glucose Conv., %	Gluconic Acid Selectivity, %	Ref.
1.	Au-SBA-15	2.1	4.9 ± 1.4	Glu/Au molar ratio = 1970, T = 383 K, t = 10 min, H_2_O_2_ = 2.2 equiv.	145,000	66	~100	This work
2.	Au-Mn(N)SBA-15	2.2	5.7 ± 1.6	Glu/Au molar ratio = 1970, T = 383 K, t = 10 min, H_2_O_2_ = 2.2 equiv.	173,000	64	~100	This work
3.	Au-Mn(A)SBA-15	2.1	6.6 ± 1.8	Glu/Au molar ratio = 1970, T = 383 K, t = 10 min, H_2_O_2_ = 2.2 equiv.	156,000	51	~100	This work
4.	Au-HBeta	1.9	7.0 ± 2.6	Glu/Au molar ratio = 1970, T = 383 K, t = 10 min, H_2_O_2_ = 2.2 equiv.	158,000	49	99	[13]
5.	Au/MgAl_2_O_4_	2.3	3.8 ± 1.0	Glu/Au molar ratio = 870, T = 393 K, t = 10 min, H_2_O_2_ = 2.2 equiv.	37,300 *	54	93	[9]
6.	Au/Al_2_O_3_	1.8	2.4 ± 0.6	Glu/Au molar ratio = 1110, T = 393 K, t = 10 min, H_2_O_2_ = 2.2 equiv.	46,200 *	83	87	[9]
7.	Au/Al_2_O_3_	0.4	2.4 ± 0.5	Glu/Au molar ratio = 27,800, T = 333 K, t = 10 min, H_2_O_2_ = 3 equiv., NaOH = 1 equiv.	312,900	>99	96	[10]

* Data from literature were recalculated according to TOF formula using in this work to obtain values which could be compared.

## Data Availability

The data presented in this study are available on request from the corresponding author via e-mail: sobiza@amu.edu.pl (I.S.).

## Supplementary Materials

The following supporting information can be downloaded at: https://www.mdpi.com/article/10.3390/ijms23094639/s1
