# Supplementary material for: Microwave-Assisted Base-Free Oxidation of Glucose with H2O2 on Gold- and Manganese-Containing SBA-15—Insight into Factors Affecting the Reaction Pathway
Source: Int J Mol Sci. 2022 Apr 22;23(9):4639. doi: 10.3390/ijms23094639 (PMC9102529; doi:10.3390/ijms23094639)
Supplement: Supplementary file 1 [file ijms-23-04639-s001.zip › ijms-1674560-supplementary.pdf]

Supplementary Materials

For Int. J. Mol. Sci.

Microwave-assisted base-free oxidation of glucose with H_2O_2 on gold- and manganese- containing SBA-15 – insight into factors affecting the reaction pathway

Izabela Sobczak, Tsering Chödon Kowalska, Magdalena Nowicka, Maria Ziolek

Adam Mickiewicz University, Poznań, Faculty of Chemistry, Uniwersytetu Poznańskiego 8, 61-614 Poznań, Poland

** corresponding author e-mail: sobiza@amu.edu.pl*

List of content:

Figure S1. Nitrogen adsorption/desorption isotherms of MnSBA-15 and Au-MnSBA-15 materials.

Figure S2. Representative TEM image of Au-Mn(N)SBA-15 showing Au NPs localization in pores of SBA-15. *Scale bare is equal to 100 nm.*

Figure S3. Gold particle size distribution histogram of Au-SBA-15 catalyst [based on: J. Wisniewska et al. *Chem. Eng. J.* **2020**, *413*, 127548].

Figure S4. FTIR spectra after (a) adsorption of pyridine at 423 K on MnSBA-15 and Au-MnSBA-15 materials and desorption at (b) 423 K, (c) 473 K, (d) 523 K, (e) 573 K. All the spectra were obtained by subtraction the spectrum after activation and normalized to the density of a wafer of 10 mg cm^{-2}.

Figure S5. FTIR spectra after (a) adsorption of pyridine at 423 K on gold modified mesoporous silica and desorption at (b) 423 K, (c) 473 K, (d) 523 K. All the spectra were obtained by subtraction the spectrum after activation and normalized to the density of a wafer of 10 mg cm^{-2}.

Table S1. Results of glucose oxidation with oxygen in pressure batch reactor over Au-Mn(N)SBA-15 and Cu-Mn(N)SBA-15 catalysts.

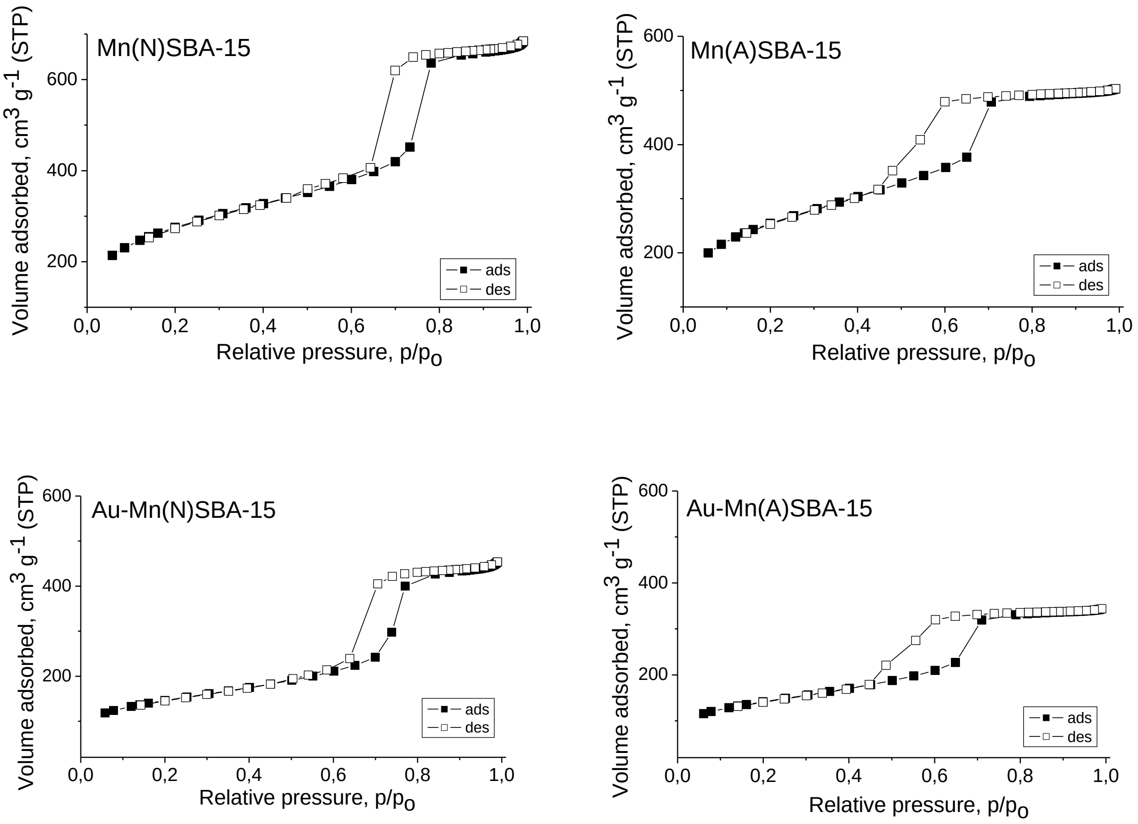

Figure S1. Nitrogen adsorption/desorption isotherms of MnSBA-15 and Au-MnSBA-15 materials.

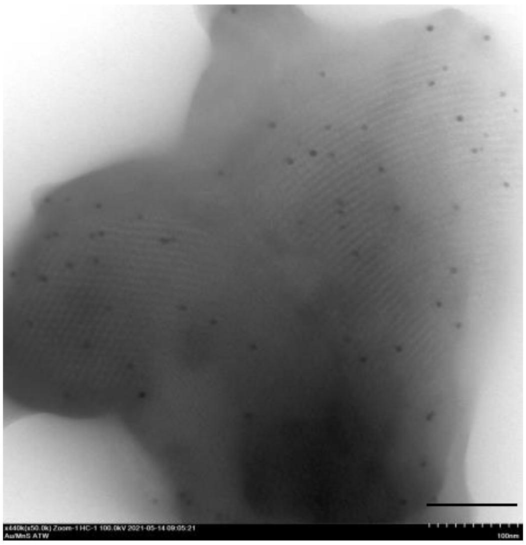

Figure S2. Representative TEM image of Au-Mn(N)SBA-15 showing Au NPs localization in pores of SBA-15. *Scale bare is equal to 100 nm.*

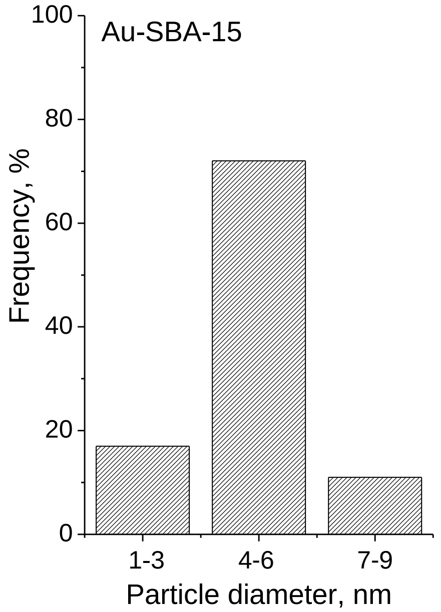

Figure S3. Gold particle size distribution histogram of Au-SBA-15 catalyst [based on: J. Wisniewska et al. *Chem. Eng. J.* **2020**, *413*, 127548].

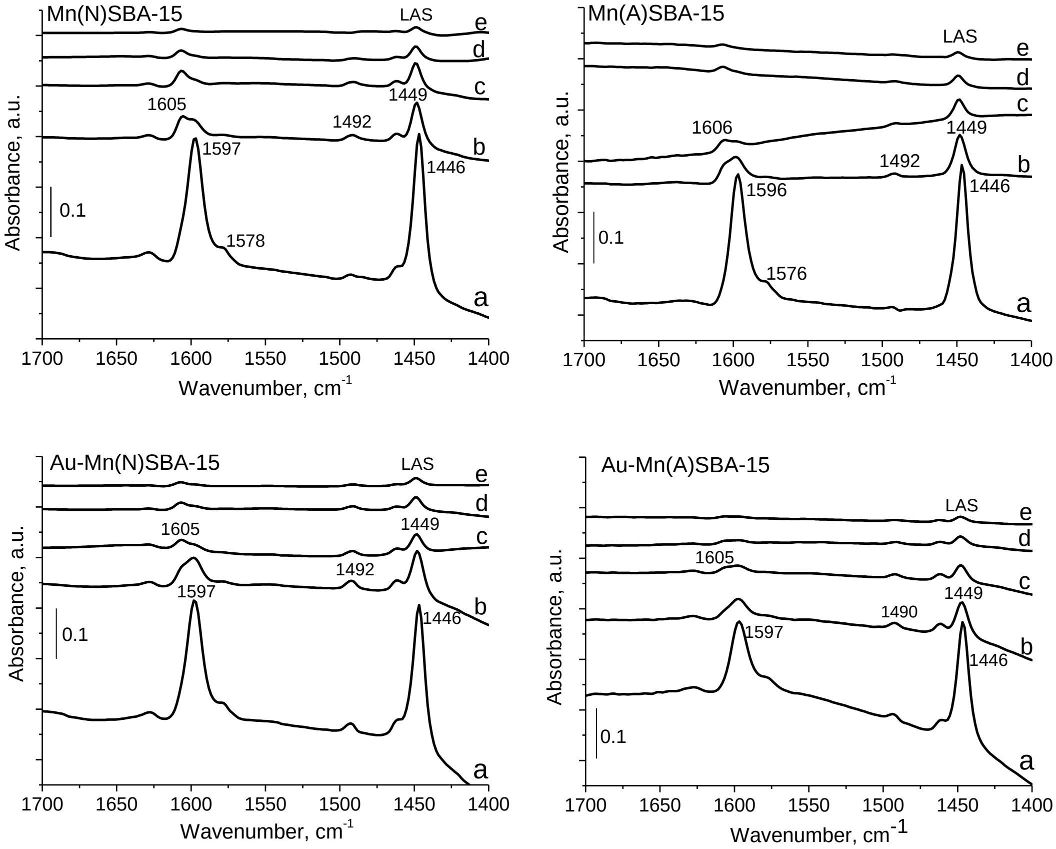

Figure S4. FTIR spectra after (a) adsorption of pyridine at 423 K on MnSBA-15 and Au-MnSBA-15 materials and desorption at (b) 423 K, (c) 473 K, (d) 523 K, (e) 573 K. All the spectra were obtained by subtraction the spectrum after activation and normalized to the density of a wafer of 10 mg cm^{-2}.

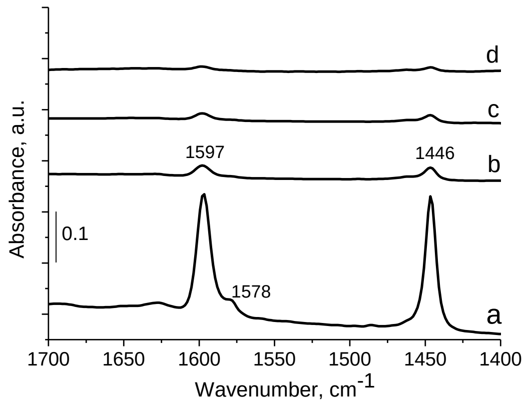

Figure S5. FTIR spectra after (a) adsorption of pyridine at 423 K on gold modified mesoporous silica and desorption at (b) 423 K, (c) 473 K, (d) 523 K. All the spectra were obtained by subtraction the spectrum after activation and normalized to the density of a wafer of 10 mg cm^{-2}.

Table S1. Results of glucose oxidation with oxygen in pressure batch reactor over Au-Mn(N)SBA-15 and Cu-Mn(N)SBA-15 catalysts.

Entry	Catalyst	Glucose conv.[a], %	Selectivity, %		TOF[b], h^{-1}
			A	B	
1.	Au-Mn(N)SBA-15	86	99.5	0.5	18,423
2.	Cu-Mn(N)SBA-15	1	traces	-	-

[a] *Reaction conditions:* 20 mL of 0.2 M glucose solution, glucose/Au (molar ratio) = 1970/1, mixing rate = 600 rpm, T = 383 K, p O_2 = 0.5 MPa, time = 120 min; A – gluconic acid, B – glucuronic acid

[b] The number of moles of gold atoms localized on the external surface of spherical Au particles was taken into account in the TOF calculations: (the number of moles of glucose converted after 2 h) × (the number of moles of gold atoms localized on the external surface of the Au NPs in a given mass of the catalyst)$^{-1}$ × h^{-1}; based on Au NPs size calculated from TEM
